# Cestode Zoonoses: Echinococcosis and Cysticercosis: An Emergent and Global Problem

**DOI:** 10.3201/eid0811.020422

**Published:** 2002-11

**Authors:** Frank O. Richards

**Affiliations:** The Carter Center, Atlanta, Georgia, USA

This book is a collection of short articles written by the participants of a research workshop held in Poznan, Poland, in September 2000. The workshop, supported by NATO Scientific Affairs, focused on the three major larval cestode diseases of humans: *Taenia solium* neurocysticercosis, *Echinococcus granulosus* cystic hydatidosis, and *E. multilocularis* alveolar hydatidosis. The format and depth of the articles are variable, but readers familiar with these parasites will find the book to be a convenient collection of new information on the subject. A shortcoming is that the book’s preface and summary are each limited to a single page. 

Perhaps most interesting for readers of the Emerging Infectious Diseases Journal are the reviews of epidemiologic data related to the emergence or reemergence of these three diseases. In sub-Saharan Africa, for example, neurocysticercosis has emerged as being more widely distributed than previously assumed and is a major cause of epilepsy. Surgery for pediatric cystic echinococcosis in Kyrgystan increased threefold during the period 1993–1998 (reaching 6 cases/100,000), suggesting new transmission probably related to worsening economic conditions after the collapse of the former Soviet Union. Surveillance for human cases of alveolar echinococcosis (which can have a mortality rate of 90% if untreated) is being strengthened in western Europe, given that *E. multilocularis* infection rates in foxes have increased in recent years. The book contains other valuable updates on diagnostics, immunology and vaccines, imaging and clinical management, geographic information systems and ecology, veterinary medicine, and community-based control programs. Readers with an interest in helminthology will find this book most useful. 

